# Health implications of racial differences in serum growth differentiation factor levels among men with obesity

**DOI:** 10.14814/phy2.70124

**Published:** 2024-12-12

**Authors:** Siresha Bathina, Virginia Fuenmayor Lopez, Mia Prado, Elliot Ballato, Georgia Colleluori, Maryam Tetlay, Dennis Tan Villareal, Sanjay Mediwala, Rui Chen, Clifford Qualls, Reina Armamento‐Villareal

**Affiliations:** ^1^ Division of Endocrinology Diabetes and Metabolism at Baylor College of Medicine Houston Texas USA; ^2^ Department of Medicine Michael E. De Bakey Veterans Affairs (VA) Medical Center Houston Texas USA; ^3^ Department of Mathematics and Statistics University of New Mexico Albuquerque New Mexico USA

**Keywords:** GDF15, Obesity, A1C, Cholesterol, Cardiovascular risk

## Abstract

Growth differentiation factor (GDF15) has been considered a biomarker and recently a hormonal driver for diseases in different populations. However, the role of GDF15 as a biomarker of health outcomes in obese men from different racial/ethnic background has not been evaluated. The objective of this study was to investigate the racial/ethnic differences on the relationship between GDF15 and markers of glucometabolic status, hormonal profile, body composition and bone mineral density (BMD) in obese men. One hundred ninety‐three obese men from diverse racial/ethnic backgrounds were enrolled. BMD and body composition were measured by dual energy X‐ray absorptiometry. Serum GDF15, osteocalcin, C‐terminal telopeptide, sclerostin, adiponectin, leptin, estradiol, testosterone, follicle‐stimulating hormone, luteinizing hormone, 25‐hydroxyvitamin D, lipid profile, and hemoglobin A1C (A1C) were measured. Non‐African Americans (NAA) had significantly higher GDF15 level than African Americans (AA). Level was also higher in patients with type 2 diabetes (T2DM). In both the groups GDF15 correlated with A1C and lean mass. However. GDF15 correlated  with body fat, LDL total cholesterol and femoral neck BMD only in NAA and with appendicular lean mass only in AA. Ethnicity, total cholesterol and T2DM were found to be independent predictors of GDF15. We conclude that GDF15 may influence glucometabolic status, body composition and bone parameters which may affect cardiovascular risk and osteoporosis  between races.

## 1 INTRODUCTION

Obesity is one of the major health concerns of this decade (Blüher, 2019; Livesey et al., 2019; Malik et al., 2013). Excess body weight is linked to a variety of health conditions including, but not limited to, increased cardiovascular (CV) risk, stroke (Kurth et al., 2002), and type 2 diabetes mellitus (T2DM; Klein et al., 2022). Recently, increased risk for fragility fractures despite a normal bone mineral density (BMD) has been added to the list of complications from obesity (Chen & Armamento‐Villareal, 2024; Gates et al., 2023). Prior studies showed racial/ethnic differences in body mass index (BMI), body fat, visceral adipose tissue (VAT) volume (Conway et al., 1995; Lovejoy et al., 1996; Perry et al., 2000), skeletal muscle mass, and BMD (Ortiz et al., 1992). Compared to Whites, Black women were reported to have low VAT, BMI, and greater appendicular skeletal muscle mass (Kanaley et al., 2003; Ortiz et al., 1992). Mortalities also vary across the different racial/ethnic groups (Woolf et al., 2018; Wong et al., 2002; Jemal et al., 2008).

In addition, racial/ethnic disparities for chronic diseases had been reported (Chang et al., 2017; Doshi et al., 2017; Ferraro et al., 2017; Kington & Smith, 1997; Lopez‐Neyman et al., 2022; Price et al., 2013). For instance, compared to Whites, African Americans were reported to have higher rates of hypertension, diabetes, and arthritis. Another study reported that Hispanics had higher rates of hypertension and diabetes but lower rates of heart disease (Kington & Smith, 1997). In addition, among those with chronic diseases, African Americans and Hispanics had worse functional status than Whites. Moreover, another study found that Blacks had higher risks of developing diabetes, hypertension, and stroke (Chang et al., 2017) with black men living shorter than white men and black women having higher life years lost than white women. Furthermore, these disparities in chronic conditions are now also observed in children (Price et al., 2013). Attempt to associate these differences to socioeconomic status found little influence of this factor on the racial variability associated with these chronic conditions (Kington & Smith, 1997), raising the possibility of a physiologic causation.

Growth differentiation factor GDF15 (also known as macrophage inhibitory cytokine 1 (MIC‐1), a member of transforming growth factor (TGF‐β) superfamily, was identified as a biomarker of aging, frailty, and metabolic disorders (Berezin, 2016; Fujita et al., 2016). GDF15 is secreted as 40 kDa pro‐peptide, which is cleaved by the endoplasmic reticulum and released into the circulation as mature GDF15 the size of 25 kDa (Li et al., 2024). Under normal physiological conditions, the circulating levels of GDF15 is <1200 pg/mL (Wiklund et al., 2010). However, marked increases in GDF15 levels were reported under conditions of cellular stress, such as patients with major cardiovascular events, and kidney failure (Li et al., 2020a; Wang et al., 2021a, b). GDF15 levels were elevated in patients with obesity (Vila et al., 2011), and these levels were further increased by the presence of T2DM (Dostálová et al., 2009). A positive association between GDF15 with A1C, triglycerides, glucose levels, and homeostatic model assessment of insulin resistance (HOMA‐IR), but negative correlation with insulin sensitivity in morbidly obese subjects with diabetes were previously demonstrated (Vila et al., 2011). In one study, high levels of GDF15 were observed in patients with atherosclerotic cardiovascular disease, heart failure, and markers of subclinical myocardial stress or injury in older adults (Echouffo‐Tcheugui et al., 2021) and these were found to influence all causes, cardiovascular and non‐cardiovascular mortality in another study (Daniels et al., 2011). Finally, GDF15 levels were positively correlated with osteoporosis in patients with thalassemia (Teawtrakul et al., 2023) suggesting its potential influence in bone health as well. Altogether these observations suggested the potential impact of GDF15 on several health outcomes.

With all the proposed influence of GDF15 on health outcomes, to our knowledge, there is very little information available if GDF15 levels could also account for the racial variability in chronic conditions. In the Longitudinal study of Aging (Semba et al., 2020) on community‐dwelling adults (22–93 years old), physical function was associated with GDF15 levels, and levels were higher in Whites compared to Blacks and Asians. However, whether physical performance varies by race and whether GDF15 has any influence has not been examined. The effect of GDF15 as a biomarker of health in relation to ethnicity in obese men has not been evaluated till date. We hypothesize that racial variability serum GDF15 levels is associated with differences in health outcomes in obese men from different racial/ethnic backgrounds. Thus, the objective of this study is to investigate the potential racial/ethnic differences on the relationship between GDF15 and markers of glucometabolic status, hormonal profile, body composition, and bone metabolism in obese men.

## 2 MATERIALS AND METHODS

### 2.1 Study design and patient population

This study is a secondary analysis of baseline data from 256 male participants with body mass index (BMI) of ≥30 kg/m^2^ aged 35–65 who volunteered for screening in two ongoing clinical trials (NCT03887936 and NCT03490513) at the Michael E DeBakey VA Medical Center (MEDVAMC) in Houston, Texas, USA. The inclusion/exclusion criteria for these clinical trials are as described previously (Russo et al., 2021, Vigevano et al., 2021). But briefly, for the first study (NCT03887936, “Testosterone and bone quality in men with diabetes and hypogonadism”) male veterans, 35–65 years old, with an average fasting morning total testosterone (T) level from 2 measurements of <300 ng/dL taken between 8 and 10 am on 2 separate days within 1 month and symptoms of hypogonadism as assessed by quantitative Androgen Deficiency in the Aging Male survey (qADAM) (Mohamed et al., 2010), having T2DM of <15 years duration, A1C of <10.5%, a fasting blood sugar of ≤180 mg/dL, BMI <35 kg/m^2^ were recruited. Diagnosis of T2DM was by history, use of medications for T2DM, A1C measurement at study entry A1C ≥6.5%, and fasting plasma glucose >125 mg/dL. Excluded were those with a history of: (1) prostate or breast cancer, (2) untreated severe sleep apnea, (3) any illness that could prevent the subject from completing the study, (4) diseases that interfere with bone metabolism (5) hematocrit of >50%, (6) prostate‐related findings on digital rectal exam, (7) serum prostate‐specific antigen (PSA) of ≥4.0 ng/mL or ≥3.0 ng/mL for African–Americans, (8) International Prostate Symptom Score (IPSS) >19, (9) on androgens, or selective androgen receptor modulators, (10) on medications affecting bone metabolism, (11) current alcohol use of >3 drinks/day, (12) history of deep vein thrombosis, pulmonary embolism, stroke or recent diagnosis of coronary artery disease, (13) a T‐score ≤−2.5 assessed by dual‐energy X‐ray absorptiometry (DXA) at the lumbar spine, total femur or femoral neck, or a history of fragility fractures (spine, hip or wrist), and/or (14) fasting total T of <50 ng/dL.

The second study, entitled “Effect of Aromatase inhibitors and weight loss in severely obese men with hypogonadism” (NCT03490513) recruits severely obese men (BMI of ≥35 kg/m^2^), 35–65 years old, with an average fasting total T done twice between 8 and 10 am on 2 separate days within 1 month of <300 ng/dL, with luteinizing hormone (LH) of <9.0 mIU/L, estradiol (E2) of ≥14 pg/mL, and with symptoms consistent with hypogonadism. The exclusion criteria are similar to the previous study, with additional exclusion such as history of: (1) clinical/biochemical evidence of hypothalamic/pituitary disease, (2) cardiopulmonary disease (e.g., myocardial infarction within 6 months, unstable angina, stroke) or unstable disease (e.g., NYHA Class III or IV congestive heart failure, severe pulmonary disease requiring steroid pills or the use of supplemental oxygen that would contraindicate exercise or dietary restriction), (3) unstable weight (i.e., ±2 kg) in the last 3 months, (4) BMD T‐score of less than −2.0 at the spine, femoral neck or total femur, (5) T2DM with fasting blood glucose of >160 mg/dL, or A1C >9.5% (Colleluori et al., 2020).

The protocols were approved by the Institutional Review Board of Baylor College of Medicine. All participants provided written informed consent in accordance with the guidelines in the Declaration of Helsinki for the ethical treatment of human subjects.

Both the studies are ongoing; NCT03887936 started in May 2018 and NCT03490513 in October 2019.

### 2.2 Body mass index (BMI)

Body weight and height were measured by a standard weighing scale and stadiometer, respectively. BMI (kg/m^2^) was calculated by dividing the weight (in kilograms) by height (in meters) squared.

### 2.3 Biochemical analyses

Blood samples were collected early in the morning after an overnight fast, processed, and then the samples were stored at −80°C until analysis. Serum total T and E2 were measured by liquid chromatography/mass spectroscopy by LabCorp Laboratory (Burlington, NC, USA), total T intra‐assay coefficient of variability (CVs) are 7.4%, 6.1%, 9.0%, 2.3%, and 0.9% at 0.65, 4.3, 48, 118, and 832 ng/dL, respectively. Inter‐assay CVs are 8.9%, 6.9%, 4.0%, 3.6%, and 3.5% at 0.69, 4.3, 45, 117, and 841 ng/dL, respectively. The detection range is 0.5–2000 ng/dL. Estradiol assay sensitivity is 0.23–405 pg/mL, intra‐assay CV is 1.4%–11.8%, and inter‐assay CV is 4.8%–10.8% (Colleluori et al., 2017) SHBG was measured with electrochemiluminescence immunoassay by LabCorp laboratory (Burlington, NC, USA). Free androgen index (FAI) was calculated as [100 × testosterone in nanograms per deciliter]/28.84 × SHBG in nanomoles per liter (nmol/L) and free E_2_ index (FEI) ([total E_2_ pg/mL] × 3.676/SHBG nmol/L) according to the methods of Sowers et al. (2008).

The following were measured by the clinical laboratory at the MEDVAMC: A1C was measured by high‐performance liquid chromatography using the Tosoh Automated Glycohemoglobin Analyzer HLC‐723G8. (Tosoh Bioscience, Inc., South San Francisco, CA, USA); triglycerides were measured by fluorometric assay, and low‐density lipoprotein (LDL) and high‐density lipoprotein (HDL) were measured by colorimetric assay by UNICEL DxC (Beckman Coulter, Inc., 250 S. Kraemer Blvd., Brea, CA 92821 USA). Detection limits for these measurements are: 11–500 mg/dL for LDL, 5–135 mg/dL (0.13–3.5 nmol/L) for HDL, 10–1000 mg/dL (0.1–11.3 mmol/L) for triglycerides; CVs 7%. Fasting glucose was measured using a Unicel DxC 800 auto‐analyzer (Beckman Coulter, Fullerton, CA, USA).

GDF15 was measured using a quantitative sandwich ELISA kit (# DGD150, R&D Systems) with intra and interassay CVs of 2.8% and 6%, respectively. The following were measured using enzyme‐linked immunosorbent assay kits: adiponectin (Millipore, EZHADP‐61K, Billerica, MA); Leptin, Human leptin ELISA kit (Millipore, EZHADP‐61 K, Billerica, MA); a C‐terminal telopeptide of type 1 collagen (CTX), a marker of bone resorption (Crosslaps; Immunodiagnostic System Inc., Gaithersburg, MD); osteocalcin (OCN), marker of bone formation (Quidel Corporation, San Diego, CA); and sclerostin (TECO Medical Sclerostin HS Enzyme Immunoassay Kit, Quidel Corp, San Diego, CA). The coefficients of variation (CVs) for the above assays in our laboratory are <10%.

### 2.4 Imaging studies

Bone mineral density (BMD) was assessed by DXA on the lumbar spine, left proximal femur (right femur if with history of prior surgery) for total femur and femoral neck regions of interest, and whole body using Hologic Discovery (Hologic Inc., Bedford, MA, USA). The CVs at our center are ~1.1% for the lumbar spine and ~1.2% for the proximal femur (Colleluori et al., 2017).

Measurement of body composition was performed using DXA (Hologic‐Discovery; Enhanced Whole Body 11.2 software version; Hologic Inc., Bedford, MA; USA). Images were analyzed according to the manufacturer's instructions. The CV for fat mass and lean mass measurements in our center is 1.5% (Aguirre et al., 2015). Visceral adipose tissue (VAT) volume (g/cm^2^) was calculated from the DXA body composition scan using APEX software (version 5.5.2; Hologic Inc., Bedford, MA) as previously described (Vigevano et al., 2021).

### 2.5 Statistical analyses

GDF15 data was not normally distributed, so it was log‐transformed for graphical purposes. Since several other variables were not normally distributed, group comparisons were performed by non‐parametric Kruskal–Wallis tests. The comparision between between African Americans (AA) and non‐African Americans (NAA) for GDF15 levels was adjusted for the presence of T2DM and T, body composition adjusted for age and T, and BMD for age and estradiol. The associations between GDF15 levels with demographic, anthropometric, body composition, bone parameters, hormonal, and metabolic parameters were analyzed by non‐parametric Spearman correlations (using ρ instead of *r*). Group differences in correlations were determined by the group‐by‐variable interaction terms in ANOVA. Since several other variables were not normally distributed, group comparisons were performed by non‐parametric Kruskal–Wallis tests. Multiple regression analysis was performed to determine the independent predictors of GDF15 levels in the entire population and separately for AA and NAA using variables that were found to be significantly different between the AA and NAA and factors that were reported to affect GDF15 levels (Dostálová et al., 2009). A *p* ≤ 0.05 is considered significant. Data were managed using Excel 2013 (Microsoft, Redmond, WA) and analyzed by Statgraphics Centurion XVI X64 (Statgraphics Technologies, Inc., The Plains, VA, USA), and results were verified using SAS 9.4 (SAS Institute, Inc., Cary, NC, USA) for tables and Graph Pad 9.0 with an unpaired parametric *t*‐test for figures. Results are presented as means ± standard deviation (SD) in tables and means ± standard error of mean (SEM) in the figures for comparison purposes.

## 3 RESULTS

### 3.1 Clinical characteristics of the study population

**FIGURE 1:**
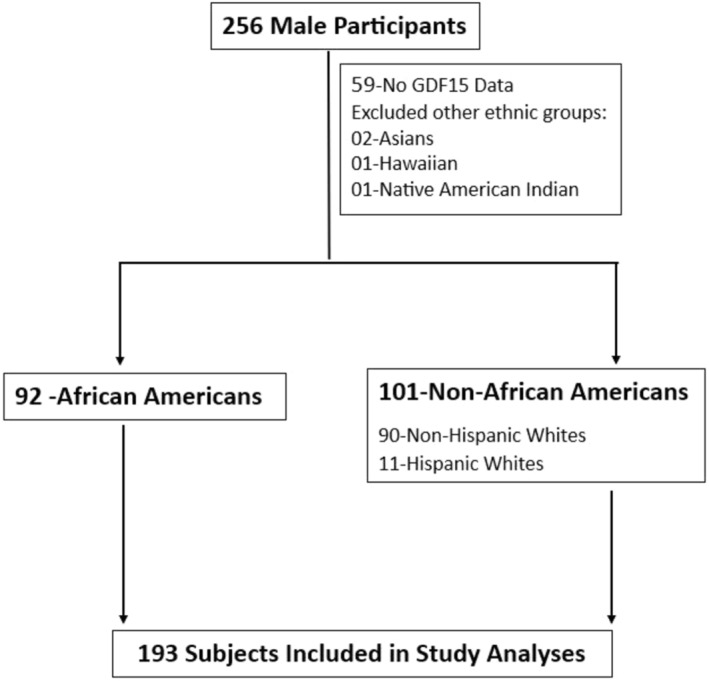
Flow diagram of enrolled participants in the study.

All 256 male subjects participated in this study of which 197 subjects have GDF15 data available, with a racial/ethnic breakdown of 92 AA, 90 Caucasians, 11 Hispanics, 2 Asians, 1 Hawaiian, and 1 Native American Indian. Following the National Institutes of Health classification of racial/ethnic groups, we grouped our subjects into AA for African‐Americans and NAA to represent both non‐Hispanic Whites and Hispanic Whites (https://www.nih.gov/nih‐style‐guide/race‐national‐origin). Because other ethnic groups were underrepresented, they were excluded in the analysis. In total, we analyzed the data of 193 subjects, 92 AA and 101 NAA (See Figure 1).

The baseline characteristics of the study population are shown in Table 1. There was no significant difference in BMI and the number of men with T2DM between the groups. Mean T was not significantly different between 2 groups. One‐hundred and five of the participants (47/92 among AA and 58/101 among NAA) had low testosterone (i.e., <264 ng/dL) according to the Endocrine Society Guidelines (Bhasin et al., 2018), but there was no significant difference in the number of patients with low testosterone between the groups. In comparison with NAA, estradiol and SHBG were significantly higher in the AA group. HDL level was significantly higher, while triglyceride was significantly lower in the AA compared to NAA. As expected, AA had significantly higher total lean mass, appendicular lean mass, and fat‐free mass compared to NAA, while NAA had significantly higher visceral adipose tissue volume than AA (Table 2). Similarly, and as expected, BMD of the lumbar spine, total hip, femoral neck, and whole body were significantly higher in AA than NAA (Table 2).

### 3.2 GDF15 levels significantly higher in NAA

**FIGURE 2:**
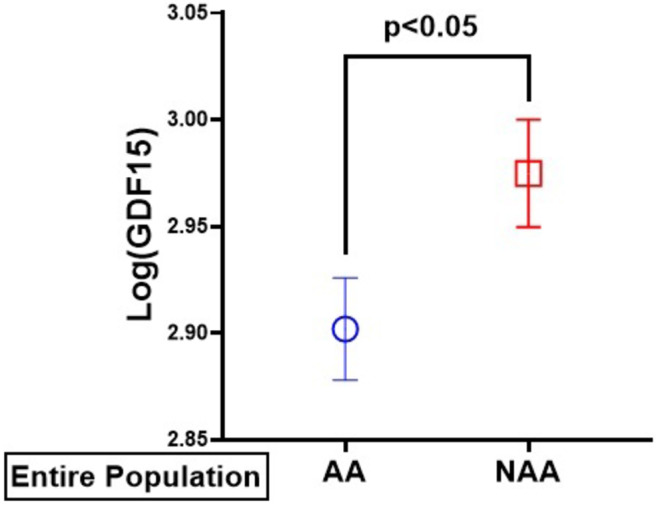
Serum growth and differentiation factor 15 (GDF‐15) (Log levels) by race Serum Log(GDF15) levels were significantly lower in African Americans (AA) compared to non‐African Americans (NAA) obese men; *p* = <0.05 unadjusted; *p* = 0.01 adjusted for T2DM, testosterone level (≤264ng/dl), age, and fat mass. Values mean ± SEM.

More importantly, comparing the two groups showed that GDF15 level was significantly higher in the NAA (1071.6 ± 65.4 pg/mL) than the AA group (908.9 ± 48.4 pg/mL); *p* = 0.03. Because the data for GDF15 were not normally distributed, we performed analysis comparing the log‐transformed GDF15 values between the 2 groups. Similarly, we observed significantly lower Log(GDF15) levels in the AA than the NAA group in both (Figure 2) unadjusted (*p* < 0.05), and after adjustment (*p* = 0.01) for T2DM, testosterone (≤264 ng/dL), age, and fat mass. The unadjusted effect size of GDF15 was 0.30 based on normalized data

**TABLE 1:** Clinical characteristics of the study population according to racial background.

Clinical outcomes	African Americans (AA)‐92	Non‐African Americans (NAA)‐105	*p*
Age(years)	53.2 ± 7.5	51 ± 7.4	0.07
BMI (kg/m^2^)	38.8 ± 5.1	39 ± 6.5	0.75
Type 2 diabetes *n* (%)	51 (55.4%)	55 (55.41%)	0.77
Testosterone (ng/dL)	281.4 ± 91.0	254 ± 103	**0.06**
Testosterone ≤264 ng/dL (%)	47 (51.08%)	58 (57.4%)	0.34
Free androgen index	36.5 ± 13.8	37.4 ± 15.03	0.73
Estradiol (pg/mL)	27.2 ± 11.5	22.5 ± 10.9	**0.006**
Free estradiol index (pmol/nmol)	3.8 ± 2.1	3.5 ± 2.0	0.36
FSH (mIU/mL)	7.52 ± 7.6	7.55 ± 6.	0.98
LH (mIU/mL)	5.13 ± 4.81	4.38 ± 2.4	0.17
SHBG (nmol/L)	29.6 ± 11.6	25.6 ± 11.4	**0.02**
25‐hydroxyvitamin D (ng/mL)	23.7 ± 10.3	26.7 ± 12.2	0.11
PTH (pg/mL)	57.9 ± 27.6	52 ± 23	0.13
CTX (ng/mL)	0.25 ± 0.13	0.22 ± 0.12	0.12
OCN (ng/mL)	5.58 ± 2.40	5.76 ± 3.39	0.70
Sclerostin (ng/mL)	0.74 ± 0.42	0.65 ± 0.30	0.08
Hemoglobin A1c (%)	7.1 ± 1.5	6.9 ± 1.5	0.51
Leptin (ng/mL)	41.2 ± 25.8	38.4 ± 23.1	0.54
Adiponectin (μg/mL)	17.7 ± 18.2	20.38 ± 23.4	0.42
Total Cholesterol (mg/dL)	170.1 ± 40.7	177.3 ± 49.47	0.28
Low density lipoprotein (mg/dL)	106.2 ± 38.1	102.4 ± 37.3	0.55
High density lipoprotein mg/dL)	41.4 ± 9.8	36.3 ± 9.04	**0.001**
Triglycerides (mg/dL)	124.3 ± 90.0	238.5 ± 235	**<0.001**

*Note*: The above table illustrates comparative analysis of hormonal and metabolic markers between AA and NAA. Free androgen index: [100 × testosterone in nanograms per deciliter]/28.84 × SHBG in nanomoles per liter and free E_2_ index ([total E_2_ pg/mL × 3.676]/SHBGnmol/L). Values are shown as mean ± SD. Bolded *p* values are statistically significant. Group comparisons were done using Kruskal–Wallis test.

Abbreviations: BMI, Body mass index; CTX‐C, terminal telopeptide of type 1 collagen; FSH, Follicle stimulating hormone; LH, Luteinizing hormone; OCN, Osteocalcin; PTH, Parathyroid hormone; SHBG, Sex hormone binding globulin.

**TABLE 2:** Body composition parameters according to racial background.

	African Americans (AA)‐92	Non‐African Americans\(NAA)‐105	*p*	Adjusted *p* *
Total body fat mass (kg)	49.30 ± 12.9	48.75 ± 14.7	0.80	0.50
Total % body fat (%)	38.8 ± 5.16	40.06 ± 5.6	0.14	0.31
Visceral adipose tissue volume (cm^3^)	1182 ± 418	1385 ± 332	**0.001**	**0.001**
Total lean mass (g)	73.1 ± 7.8	69.2 ± 7.0	**0.008**	**0.001**
Appendicular lean mass (kg)	33.2 ± 4.1	30.8 ± 6.0	**0.006**	**0.005**
Fat‐free mass (kg)	74.2 ± 13.7	71.8 ± 7.2	**0.02**	**0.026**
Lumbar spine (g/cm^2^)	1.20 ± 0.17	1.1 ± 0.16	**0.001**	**<0.001**
Total hip (g/cm^2^)	1.16 ± 0.13	1.1 ± 0.13	**0.002**	**0.002**
Femoral neck (g/cm^2^)	1.0 ± 0.14	0.9 ± 0.13	**<0.001**	**<0.001**
Whole body (g/cm^2^)	1.2 ± 0.13	1.1 ± 0.09	**<0.001**	**<0.001**

*Note*: The above table illustrate comparative analysis of body composition markers between AA and NAA. Values are shown as mean ± SD. Bolded *p* values are statistically significant.

* *p* adjusted for age and testosterone for body composition, testosterone and estradiol for bone mineral density. Group comparisons were done using Kruskal–Wallis test.

### 3.3 Correlations with metabolic and hormonal parameters

Table 3 shows the correlations between GDF15 and hormonal and metabolic variables. GDF12 positively correlated with age and A1C, and negatively with BMI in both the racial groups. It also correlated positively with FSH and LH, but negatively with PTH, adiponectin, total, and LDL cholesterol only in the NAA group. On the other hand, it correlated negatively with estradiol, CTX, and leptin only in the AA group.

**TABLE 3:** Correlations between GDF 15 with Clinical, hormonal, and Metabolic Parameters.

	African Americans (AA)‐92		Non‐African Americans (NAA)‐101	
	*p*	*p*	*p*	*p*
(A) Demographic and Chemical parameters				
Age (years)	0.26	**0.01**	0.39	**<0.001**
BMI (kg/m^2^)	−0.21	**0.04**	−0.34	**<0.001**
Testosterone (ng/dL)	−0.07	0.49	0.12	<0.25
Free androgen index	0.09	0.39	0.14	0.17
Estradiol (pg/mL)	−0.26	**0.01**	−0.09	0.37
Free estradiol index (pmol/nmol)	−0.03	0.76	0.004	0.96
FSH (mIU/mL)	0.03	0.78	0.36^a^	**<0.001**
LH (mIU/mL)	0.20	0.06	0.30^a^	**0.002**
SHBG (nmol/L)	−0.15	0.16	−0.05	0.63
25‐hydroxyvitamin D (ng/mL)	−0.03	0.78	0.17^a^	0.09
PTH (pg/mL)	−0.14	0.26	−0.23	**0.03**
CTX (ng/mL)	−0.38	**0.001**	−0.20	0.06
OCN (ng/mL)	−0.01	0.40	−0.09	0.36
Sclerostin (ng/mL)	−0.02	0.265	0.02	0.82
Hemoglobin A1C (%)	0.43	**<0.001**	0.40	**<0.001**
Leptin (ng/mL)	−0.30	**0.006**	−0.21	0.06
Adiponectin (μg/mL)	−0.16	0.16	−0.31	**0.003**
Total Cholesterol (mg/dL)	−0.20	0.08	−0.28	**0.004**
High density lipoprotein (mg/dL)	0.01	0.93	−0.08	0.40
Low density lipoprotein(mg/dL)	−0.21	0.06	−0.30	**0.017**
Triglycerides (mg/dL)	0.004	0.97	0.02	0.81
(B) Body composition				
Total body fat (kg)	−0.01	0.38	−0.27	**0.009**
Total % body fat (%)	−0.03	0.77	−0.27	**0.01**
Visceral adipose tissue vol (cm^3^)	−0.04	0.71	−0.10	0.33
Total lean mass (g)	−0.24	**0.03**	−0.25	**0.02**
Appendicular lean mass (kg)	−0.23	**0.05**	0.02	0.83
Fat‐free mass (kg)	−0.18	0.09	−0.25^a^	**0.01**
(C) Bone mineral density				
Lumbar spine (g/cm^2^)	−0.08	0.46	0.06	0.59
Total hip (g/cm^2^)	0.02	0.85	−0.18	0.09
Femoral neck (g/cm^2^)	−0.02	0.83	−0.32^a^	**0.002**
Total body (g/cm^2^)	−0.07	0.55	0.07	0.51

*Note*: Bolded *p* values are statistically significant.

Abbreviations: BMI, Body mass index; CTX‐C, terminal telopeptide type 1 collagen; FSH, Follicle stimulating hormone; LH, Luteinizing hormone; OCN, Osteocalcin; PTH, Parathyroid hormone; SHBG, Sex hormone binding globulin.

^a^ *p* ≤0.05 indicates a significant difference between the two correlations, this test was done by a group‐variable interaction in ANOVA.

### 3.4 Correlations between GDF15 with body composition

For body composition (Table 3), significant correlations for GDF15 were found, that is, negatively for total lean mass in both groups; negatively for total % body fat, total body fat mass (kg), and fat‐free mass only in the NAA; and negatively for appendicular lean mass only in AA.

### 3.5 Correlations between GDF15 with bone mineral density

With regards to BMD, GDF15 significantly negatively correlated with femoral neck BMD only in the NAA group (Table 3). There was no significant correlation between GDF15 with any other BMD parameters in both groups. A comparison of the correlations using group‐variable interaction in ANOVA showed that the correlations between GDF15 and factors such as FSH, LH, vitamin D, fat‐free mass, and femoral neck BMD were significantly different between AA and NAA.

### 3.6 Independent predictors for GDF15

Results from our analyses on the independent predictors of GDF15 levels using multiple regression model, including ethnicity as one of the variables, are shown in Table 4. In the entire population, total cholesterol, ethnicity (AA) and T2DM were significant independent predictors of GDF15 levels. In the AA group, T2DM was the only independent predictor found, while total cholesterol, fat‐free mass, T2DM and FSH were found to be independent predictors of GDF15 in the NAA group. All models were adjusted for age. A statistical multiple regression model including ethnicity as one of the variables is elaborated in Table 4. In the entire population, total cholesterol, ethnicity (AA), and T2DM were significant independent predictors of GDF15 levels. In the AA group, T2DM was the only independent predictor found, while total cholesterol, fat‐free mass, T2DM, and FSH were found to be independent predictors of GDF15 in the NAA group. All models are adjusted for age.

**TABLE 4:** Multiple regression of GDF15 by ethnicity and the entire population.

	*R* ^2^	Beta estimate	SE	*p*
GDF15 (Entire population) (*N* = 188)	0.27			
Total Cholesterol (mg/dL)		−2.74	0.83	**0.001**
Ethnicity (AA)		−215.03	74.06	**0.004**
T2DM		402.01	76.81	**<0.001**
AA group (*N* = 90)	0.19			
T2DM		365.5	89.8	**0.005**
NAA group (*N* =881)	0.39			
Total Cholesterol (mg/dL)		−2.78	1.31	**0.04**
Fat free mass (kg)		−0.019	0.008	**0.02**
T2DM		417.8	127.8	**0.002**
FSH (mIU/mL)		31.83	10.29	**0.003**

*Note*: The table above illustrates multiple regression models using ethnicity as one of the variables. Bolded *p* values are statistically significant. All models adjusted for age.

**FIGURE 3:**
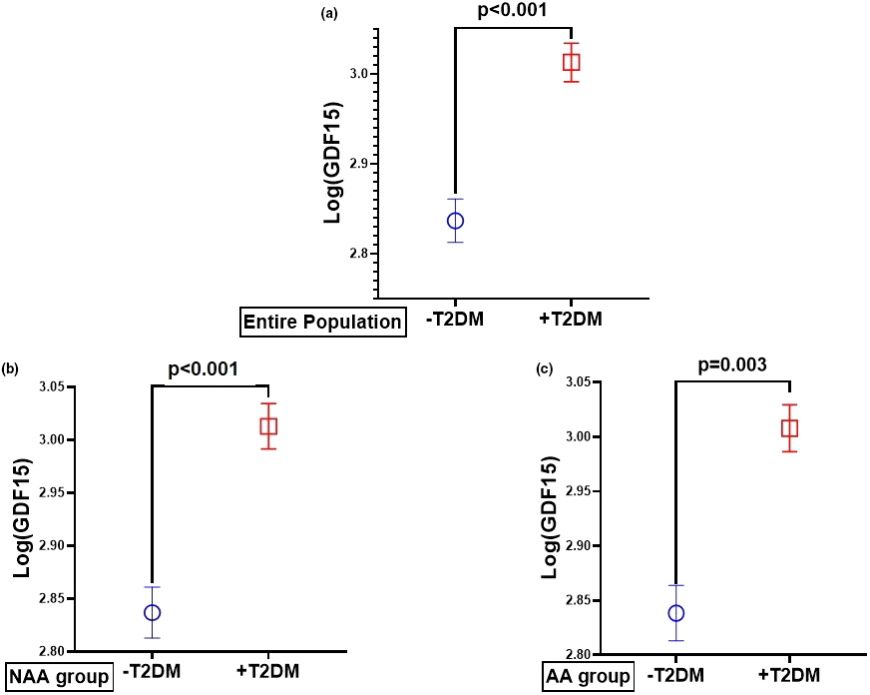
Serum Log (GDF15) levels according to the presence of type 2 diabetes mellitus (T2DM) for the entire population, and by race or ethnicity. Log(GDF15) levels were significantly higher in a) entire population with T2DM p<0.001, b) African Americans (AA) with T2DM, *p* = 0.003, and c) Non‐African Americans (NAA) with T2DM *p* < 0.001, when compared to the non‐diabetic group(‐T2DM). All values mean ± SEM.

We also analyzed our subjects into those with and without T2DM. We found that Log(GDF15) levels were significantly higher in patients with T2DM in the entire population (Fig. 3a). A separate analysis according to race showed that those with T2DM in the NAA (Fig. 3b) and AA (Fig. 3c) had significantly higher Log(GDF15) levels than those without T2DM. We also analyzed our subjects into those with and without T2DM. We found that GDF15 levels were significantly higher in patients with T2DM (Figure 2b). A separate analysis according to race showed that those with T2DM in the NAA (Figure 2c) and AA (Figure 2d) had significantly higher GDF15 levels compared to those without T2DM.

## 4 DISCUSSION

GDF15 is a molecule secreted by most cell types in the body in response to cellular or tissue stress resulting from a variety of causes such metabolic, inflammatory, cardiovascular, and malignant disorders (Asrih et al., 2023; Berezin, 2016; Breit et al., 2021; Wang et al., 2021a, b). The associations between GDF15 with obesity (Valenzuela‐Vallejo et al., 2022) diabetes, (Niu et al., 2021), cardiovascular disease (Kempf et al., 2007; Li et al., 2020a; Peiró et al., 2019), and recently osteoporosis (Li, et al., 2020b; Mattia et al., 2023; Osawa et al., 2022) highlight the significance of GDF15 in human health. However, no study has yet established a link between GDF15 and the varying risks for these conditions among races, more especially in the racially diverse obese population. Racial variability in GDF15 levels had been reported in the Baltimore Longitudinal Study of Aging (Semba et al., 2020) showing that Whites had higher levels compared to Blacks and Asians. This study also showed an association between GDF15 and sarcopenia (Semba et al., 2020) but whether there was racial diversity in sarcopenia risk in relation to GDF15 levels was not reported. In our study, we observed significantly higher GDF15 levels in NAA than AA which correlated with the different health outcomes showing racial variability for certain measures.

Obesity, which is associated with inflammation (Leisegang et al., 2019), is commonly accompanied by T2DM (another pro‐inflammatory state) (Rohm et al., 2022). In a cohort study, it was reported that those who were morbidity obese (*N* = 118) had significantly higher GDF15 level compared to sex‐matched lean control subjects (*N* = 30), but the highest concentration was seen among those with coexisting T2DM (Vila et al., 2011). Positive associations between GDF15 with triglycerides, A1C, glucose, and HOMA‐IR, and a negative correlation with insulin sensitivity (Vila et al., 2011) were also observed; suggesting a negative influence of GDF15 on glucometabolic parameters. Similarly, we found a positive association between GDF15 and A1C in both racial/ethnic groups. T2DM subjects also had significantly higher GDF15 levels compared to non‐T2DM regardless of race. However, unlike the previous study, we found negative associations between GDF15 with total and LDL cholesterol in the NAA group. Furthermore, total cholesterol remained a negative independent predictor of GDF15 levels in the entire population and in NAA. These latter findings were in agreement with the findings from Vitamin D‐systolic hypertension (VitDISH; Barma et al., 2017) and in the Rancho Bernardo studies showing negative correlations between GDF15 with total and LDL cholesterol (Daniels et al., 2011). In the Rancho Bernardo study, GDF15 was a strong predictor of CV, non‐CV, and all‐cause mortality, with those in the highest quartile having the highest risk. The negative correlation between cholesterol and GDF15 levels and the consistent increase in CV risk associated with high GD15 would suggest that GDF15 influences CV risk independently of other risk factors. Indeed, evidence from PIVUS study (Lind et al., 2009) showed that high GDF15 was associated with higher prevalence of CV disease independent of traditional CV risk factors such as male gender, BMI, waist circumference, diabetes, fasting glucose, triglycerides, and low HDL. The above data would suggest that GDF15 could be a biomarker to follow‐up or predict CV risk and mortality outcomes. (Adela & Banerjee, 2015). Because a history of CV disease was for the most part an exclusion criterion, we were unable to assess the relationship between CV risk and GDF15 levels in the relatively healthy obese men in our study.

While several studies showed a positive correlation between GDF15 and BMI (Dostálová et al., 2009; He et al., 2020; Sarkar et al., 2020), our study showed the opposite. By comparison, these prior studies were done in subjects over a wider range of BMI which included not only obese subjects, but also those with normal and overweight BMI, and had both men and women as subjects. Our population were mainly obese men with a BMI range of 30.3–55.8 kg/m^2^ among AA and 30.2–62.7 kg/m^2^ among NAA subjects. In addition, most of the subjects in our study had low testosterone, that is, 105 out of 193 participants for the entire population, 47 out of 92 in AA and 58 out of 101 in NAA. Nevertheless, other studies also showed a negative correlation between BMI and GDF15 level. In a group of 142 subjects with chronic obstructive pulmonary disease with BMI ranging from 17.56 to 25.8 kg/m^2^, BMI was negatively correlated with GDF15 (−0.562, *p* < 0.001; Shi et al., 2024). Another study on a group of 93 subjects (which included both healthy and those with impairment in lower extremity mobility) also showed a negative correlation between BMI and GDF15 (*r* = −0.26) although the significance was borderline (*p* = 0.081); Chiariello et al., 2024). Moreover, a study among 72 monozygotic nonobese twins, ranging in age between 63‐93 years old of which 69% were female pairs, showed that the twin of the twin pair with the higher serum GDF15 level generally had lower BMI than their identical twin pair, and vice‐versa (Tsai et al., 2015). Finally, GDF15 was also found to negatively correlate with BMI in pregnant women (Petry et al., 2018). Hence, we surmise that the discrepancy in the relationship between BMI and GDF15 could be a function of the population under investigation.

A study reported that reduced muscle mass and impaired muscle function accompanied high levels of GDF15 in aged male C57/B6 mice, and in older women (Kim et al., 2020). Additionally, Oba et.al. reported that high GDF15 was associated with poor muscle strength in 257 older patients with cardiometabolic disease (Oba et al., 2020). Similarly, we observed a negative correlation between GDF15 with total lean mass in both groups, with fat‐free mass in NAA, and appendicular lean mass in AA. Moreover, fat free mass was an independent negative predictor of GDF15 levels in NAA further supporting a negative association between lean mass and GDF15. Unfortunately, we did not evaluate the muscle function in our subjects. Interestingly, a preclinical study (Wang et al., 2023) showed that GDF15 administration in pharmacologic doses to calorically‐ restricted mice induced further weight loss beyond 10 days compared to pair‐fed mice mainly through to fat loss since these mice did not loss lean mass. Long‐term weight loss is usually not maintained despite continued caloric restriction mostly due to physiological adaptation that suppresses energy expenditure, a process known as adaptive thermogenesis (Nunes et al., 2022). In the above study, GDF15 administration was able to counteract this reduction in energy expenditure by maintaining energy expenditure in the skeletal muscle leading to a more sustained longer‐term weight loss (Wang et al., 2023).

Also, in our study, NAA subjects who had significantly higher GDF15 levels had significantly higher VAT volume than AA subjects. Furthermore, an inverse correlation between GDF15 and body fat was observed in NAA suggesting that perhaps GDF15 may negatively regulate body fat in NAA. Gonadal hormones which decline with age also influence cardiometabolic risks (Baumgartner et al., 1999; Jung et al., 2020). We found a significant positive association between pituitary hormones FSH and LH with GDF15 in NAA but no association between testosterone and estradiol with GDF15 in this group. On the other hand, GDF15 negatively correlated with estradiol in the AA group although no correlation was found for FSH and LH in this group. However, by multiple regression analysis, we found that FSH independently influenced GDF15 in NAA group. Report of GDF15 providing endocrine signals to the pituitary (Patel et al., 2019) in particular to the hypothalamic‐pituitary‐adrenal (HPA) axis in response to stressful stimuli with subsequent increase in circulating glucocorticoids had been published (Cimino et al., 2021). Yet, information on the converse of pituitary hormones regulating GDF15 levels remains unknown.

GDF15 was also reported to uncouple bone formation from resorption in myeloma patients with osteolytic bone disease (Westhrin et al., 2015). Limited studies in humans suggested a negative influence of GDF15 on bone but mostly in women (Kralisch et al., 2020; Morton et al., 2003). It was found to negatively correlate with BMD at the lumbar spine, total hip, and femoral neck in postmenopausal Chinese women (Li, et al., 2020b). Our results showed a significant negative correlation between bone resorption marker CTX and GDF15 levels in AA; however, its influence on BMD was only observed in the femoral neck on NAA. A similar finding was reported in a group of postmenopausal women of Korean ancestry (Lee et al., 2022). Thus, the possibility that high GDF15 levels may be a risk factor for a hip fracture needs further investigation.

Although GDF15 has traditionally been thought of as a biomarker, with the recent discovery of glial cell line‐derived neurotrophic factor family receptor alpha like (GFRAL) in the brainstem, it is now considered as a hormonal driver (Lockhart et al., 2020). It has been implicated as part of the mechanisms responsible for a host of disorders involving cardiac, vascular, metabolic, neurodegenerative, ocular, and tumor pathologies (Rochette et al., 2020). Furthermore, because of the anorectic effect of GDF15, it has become a potential therapeutic target in the management of obesity, metabolic syndrome (Wang et al., 2021a, b) cancer related anorexia (Assadi et al., 2020; Borner et al., 2020), and cachexia (Ling et al., 2023). Finally, its effect on sparing lean mass during long‐term weight loss in calorically restricted mice (Wang et al 2023), if confirmed in humans, is a potential advantage of GDF15 over current FDA‐approved weight loss medication which are associated with loss of lean mass (Neeland et al., 2024). Studies directed at modifying GDF15 levels, either by pharmacologic means or lifestyle means, are necessary to determine if doing so will alter risk for certain diseases.

The strength of our study is that we are the first to report the differences in GDF levels between AA and NAA, and its relationship with biomarkers of glucometabolic status, hormonal profile, body composition, and bone metabolism in a relatively large number of middle‐aged obese men from different racial/ethnic backgrounds. However, our study has several limitations. Firstly, it is cross‐sectional in nature; therefore, we cannot infer the cause‐and‐effect relationship between GDF15 and the health outcomes of interest. Secondly, since our population consists of obese men among whom low testosterone is prevalent, our results cannot be applied to the general population of men and women from different racial background. On the other hand, given the potential for GDF15 to mediate a host of conditions, measuring levels of this important molecule in populations which are often overlooked but may have high levels of disease is of great consequence. If drug development targeting GDF15 signaling becomes successful, identifying those who would be responsive to the therapy based on GDF15 levels would direct treatment to this group of patients. Finally, although our results are derived from associative analysis and therefore do not establish causation, in most cases hypotheses are often generated from association studies.

In summary, we found that among obese men, GDF15 levels vary according to racial/ethnic background; it was significantly lower among AA than NAA. Furthermore, GDF15 levels were significantly higher in those with T2DM regardless of race. In terms of metabolic markers, GDF15 positively correlated with A1C in both groups but negatively with total cholesterol and LDL only in NAA. Moreover, GDF15 levels negatively correlated with total lean mass in the entire population, with appendicular lean mass in AA, and total body fat, and fat free mass in NAA. However, for bone, the only correlation found was in the femoral neck (i.e., negative) in NAA. Thus, GDF15 may in part influence or be influenced by certain cardiometabolic risk factors such as diabetes mellitus and A1C in both racial groups. Given its negative association with LDL and total cholesterol, the reported increased CV risk with high GDF15 levels was likely independent of lipid levels at least in the NAA group.

## AUTHOR CONTRIBUTIONS

Conceptualization: S.B. and R.A.V.; Formal analysis: S.B., C.Q., and R.A.V.; Investigation: S.B., V.F.L., M.P., E.B., G.C., M.T., D.T.V., S.M., R.C., and R.A.V.; Writing: S.B., C.Q., and R.A.V.; reviewing and editing: S.B., V.F.L., M.P., E.B., G.C., M.T., D.T.V., S.M., C.Q., R.C., and R.A.V. All authors have read and agreed to the published version of the manuscript.

## FUNDING INFORMATION

This study was supported by VA merit review 101CX000424, 101CX001665, and NIH R01 HD093047 to R.A.V. The findings reported in this article are the result of work supported with resources and the use of facilities at the New Mexico VA Health Care System and Michael E. De Bakey VA Medical Center.

## CONFLICT OF INTEREST STATEMENT

The authors declare no conflicts of interest.

## DISCLOSURE

Nothing to disclose

## ETHICS APPROVAL AND CONSENT TO PARTICIPATE

All patients provided written informed consent for publication of their clinical details was obtained before the beginning of the study.

## Data Availability

The datasets used and/or analyzed during the current study are available from the corresponding author on reasonable request.
